# Changing predictive asymmetry: network agenda correspondence between state media and the user on China's fertility policy (2013–2025)

**DOI:** 10.3389/fpubh.2026.1943400

**Published:** 2026-10-16

**Authors:** Wen Deng, Zijun Mao, Yi Yang

**Affiliations:** 1 School of Journalism and Culture Communication, Zhongnan University of Economics and Law, Wuhan, China; 2 Non-traditional Security Research Center, Huazhong University of Science and Technology, Wuhan, China; 3 College of Public Administration, Huazhong University of Science and Technology, Wuhan, China; 4 School of Public Administration, Guangdong University of Finance and Economics, Guangzhou, China

**Keywords:** agenda-setting, family planning policy, public opinion, social media, Weibo

## Abstract

**Introduction:**

Over the past decade, China's fertility policy has shifted from restrictive population control toward pronatalist support, creating an important case for examining the adaptation of government communication in a platformized media environment.

**Methods:**

We analyzed 126 fertility-policy posts published by *People's Daily* and 196,218 associated user comments on Weibo between 2013 and 2025. The LDA model was used to identify four topic-derived policy attributes. Event-based cross-lagged correlations evaluated directional associations against the Rozelle-Campbell baseline, while topic joint-intensity networks and event-level QAP correlations assessed aggregate structural correspondence. Analyses were conducted across three periods: the selective two-child reform period, the universal two-child policy period, and the three-child and pronatalist-support period.

**Results:**

Four topic-derived attributes emerged: *Policy Change and Childrearing Burdens, Women's Employment and Work-Family Responsibilities, Gender Preference and Family Norms*, and *Maternity Care and Childbirth Support*. In the first two periods, media-preceding-only patterns were descriptively more frequent than user-preceding-only patterns. In the third period, user-preceding-only patterns became more frequent. Gender analysis revealed gender- and period-specific differences in users' relative attention to the four policy attributes.

**Discussion:**

The relationship between the state-media and user agendas was heterogeneous rather than consistently media-led or user-led. Aggregate thematic similarity could coexist with weaker event-level structural correspondence. The findings are consistent with a potentially more reciprocal communication environment in the later policy period. It also indicates that fertility-policy communication should recognize gender-differentiated issue attention within the user agenda.

## 1 Introduction

Population policy is a critical determinant of national development and familial wellbeing (1). For decades, China's state-led family planning regime operated under a coercive one-child policy framework, legitimized as a “basic national policy” essential for modernization (2). Within this centralized propaganda architecture, policy compliance was framed as a patriotic contribution to national development (3), while alternative narratives faced structural marginalization (4). This ideological mediation facilitated the internalization of fertility limitations and constrained overt contestation, effectively embedding family planning within broader nation-building discourses (5–7).

Over the past decade, mounting concerns over population aging and demographic decline have prompted a fundamental reorientation of China's fertility policy. The policy trajectory has evolved through three distinct legislative milestones: the selective two-child policy adopted at the Third Plenum of the 18th CPC Central Committee in November 2013, permitting couples to have a second child if one parent was an only child; the universal two-child policy announced at the Fifth Plenum in October 2015 and formally enacted into law in January 2016; and the three-child policy, first deliberated by the Politburo on May 31, 2021, and formally promulgated comprehensive pronatalist supports on June 26, 2021 (8–11). This trajectory further extended to universal childbirth subsidies and preschool fee waivers by 2025 (12). This unprecedented policy shift represents not merely a series of incremental legislative adjustments but also a major strategic communication challenge, as authorities have had to repeatedly recalibrate public messaging to construct a “birth-friendly” institutional environment across three distinct and non-overlapping policy phases.

Concurrently, this policy inflection has unfolded alongside the platformization and pluralization of China's media ecology (13). The rise of social networking and “self-media” platforms has weakened the state's previous messaging monopoly, fostering more heterogeneous and contested public discourse that may contribute to how fertility policies are interpreted and evaluated by the users. As official agenda-setting increasingly intersects, and at times collides, with bottom-up interpretations, the distribution of public opinion has become an integral dimension of contemporary demographic policy communication (4, 14, 15).

Yet the precise nature of this intersection remains empirically underspecified. It remains unclear whether state-media agendas continue to precede and correspond with subsequent user discussion, whether public concerns increasingly precede subsequent official attention, or whether the relationship has developed into a more reciprocal pattern of temporal correspondence across policy stages. Resolving this question requires more than a static comparison of what topics each side discusses; it calls for a longitudinal account of how attribute salience and the linkages among attributes evolve in tandem, or diverge, as policy moves from initial relaxation to full pronatalist mobilization.

Against this backdrop, the study maps the evolution of online interactive discourse on China's population policy across the 2013–2025 reform sequence. Employing comparative agenda analysis of state-run media outputs (as proxies for the official agenda) and user-generated social media content (the user agenda), it delineates shifting attribute salience, thematic frames, and evaluative stances across the state-media and user agendas, and traces points of consensus and dissensus at each policy inflection. The remainder of this article proceeds as follows: Section 2 reviews relevant literature on China's media governance, social media affordances, and fertility policy discourse. Section 3 details our data collection procedures, text mining pipeline, and network analytical methods, through which we identify the salient attributes of fertility policy discourse, examine the direction and relative strength of event-lagged agenda associations using cross-lagged correlation (CLC) analysis anchored to the Rozelle-Campbell baseline, and trace the structural evolution of media-user attribute networks across the three policy periods using QAP analyses. Section 4 presents our findings regarding attribute identification, agenda-setting dynamics, network convergence, and gender differences. Section 4 presents the results concerning topic-derived attributes, event-lagged agenda correspondence, network correspondence, and gender differences. Section 5 discusses the theoretical and substantive implications of the findings. Section 6 concludes the study, and Section 7 outlines its limitations and directions for future research.

## 2 Related work

Within China's media governance architecture, state-run news organizations function as agents of the authorities, producing politically consonant content and sustaining a predominantly pro-government information environment (16, 17). During the one-child policy era, this centrally managed propaganda system positioned state media not merely as a passive “mouthpiece,” but as an active amplifier within the policy implementation chain, shaping perceptions, normative expectations, and behavioral compliance (18). For instance, Zhang and Yuan (19) find that access to mass media significantly increased household adherence to the family planning policy in the 1980s.

The diffusion of the commercial Internet and social networking sites (SNS) has reconfigured patterns of information access, circulation, and interpretation, enabling citizens to deconstruct, remix, and contest official narratives (20). Confronted with a pluralized, platformized, and algorithmically mediated attention economy, legacy state outlets have established social media presences to retain agenda-setting capacity and symbolic authority (21). SNS can be regarded as a quasi-Habermasian public sphere (2, 4, 22). The result is a shift from a vertically integrated propaganda paradigm toward a hybrid communicative ecology in which state-run media must negotiate resonance amid diversified publics.

Meanwhile, SNS furnish Chinese authorities with interactive channels for sentiment monitoring, issue framing, and early risk detection, functioning as a governance “safety valve” (23). They have been institutionalized as instruments of digital public management and propaganda, enabling targeted, future-oriented framing (24). Regarding China's family planning policy in media discourse, Dai and Jiang (25) show that state media acknowledged pressures (rising healthcare demand, deteriorating working conditions for women), while assuring their eventual resolution and projecting demographic and economic gains. By contrast, bottom-up discourse problematizes population policy as a multidimensional socio-political issue, linking sustained fertility decline to reproductive rights, gender inequality, childcare burdens, and perceived policy inconsistency (17), thereby exposing a structured gap between solutionist official narratives and experiential public critique. Upon the announcement ending the three-decade, strictly enforced one-child policy, intense debate predictably surfaced on SNS, where the expanded online sphere disrupts the state's former near-monopoly over public discourse (4, 22). Simultaneously, SNS infrastructures lower dissemination costs, accelerate timeliness, and enable reciprocal, albeit constrained, interaction accountability between authorities and citizens (26).

An analysis of comments on the two-child policy (2013–2019) identifies four recurrent points of contention: women's right to work, family culture and tradition, law and regulation, and social welfare and wellbeing (14). Following the 2021 announcement of the three-child policy, supportive users framed the shift as an expansion of reproductive choice, whereas dissenting voices emphasized enduring structural constraints: simultaneous responsibilities for child and elder care, high housing and child-rearing costs, rising eldercare demands, gendered labor-market penalties, persistent gender-essentialist norms, pessimistic job prospects, and an ironic skepticism about the policy's problem-solving capacity (27–29). Further research has found that, although online discourse surrounding the three-child policy exhibits opinion polarization, sustained expression and cross-group interaction can partially depolarize views (22). Wang et al. (30) conducted a dynamic monitoring of public opinion on fertility intentions, the public attitudes were positive during the period of 2015–2016, while from 2020 to 2023, fertility intentions significantly declined, with neutral and negative emotions gradually dominating.

In the social media era, different and often opposing social groups use networked communication to construct their own, sometimes conflicting, versions of public opinion (4). Debates over family planning policy provide a vantage point for observing how a pluralistic public sphere showcases heterogeneous actors. Within this discursive arena, the gendered individual serves as a salient categorical frame. Deng et al. (14) show that female SNS users foreground “female employment rights,” whereas male users emphasize “policy and legislative frameworks.” Han (6) further finds that women are most concerned with the financial burden of simultaneously caring for young children and aging parents. Female users expose the social inequalities shaping their family and professional lives and express aversion to, and resistance against, pronatalist pressures on SNS.

This body of gender-focused research illustrates a broader pattern in how family-planning discourse has been studied to date: scholars have developed a rich array of qualitative and computational tools, yet applied them largely to describe one side of the conversation at a single moment in time, rather than to compare how media and public discourse evolve together. Critical discourse analysis has been used to unpack the ideological framing embedded in official rhetoric (28, 31, 32), framing analysis has traced how specific metaphors and narrative frames shift across policy announcements (33), and semantic network analysis has mapped the keyword co-occurrence structure within a given text corpus to reveal dominant discursive clusters (4, 34). Each approach offers valuable insight into a specific discursive actor or a bounded time window, but typically stops short of directly comparing media and public discourse against one another, or of tracking how their respective attribute structures change across successive policy stages. Given the dynamic, multisource, and interactive nature of issue presentation on social networking sites, this single-actor, single-snapshot focus offers only a partial account of how issues are jointly constructed and how their communicative effects unfold over time.

Compared to previous studies, to capture how fertility policy attributes are cognitively linked rather than merely how often they appear, this study adopts the Network Agenda-Setting (NAS) approach (35). Unlike conventional agenda-setting analysis, which compares the salience of individual issues or attributes between media and public discourse, NAS treats attributes as nodes in a network and examines how their co-occurrence patterns, for instance, which attributes are discussed together and whether similar patterns of attribute linkage appear across media and public texts (36–38). This framework provides a data-driven way to examine whether patterns in one agenda temporally precede and predict corresponding patterns in the other at different stages of policy implementation, offering a longitudinal, relational account of how state-media and user fertility-policy agendas develop in relation to one another across distinct policy periods.

## 3 Materials and methods

### 3.1 Data sources and pre-processing

Weibo is one of China's largest SNS platforms. Functioning as an open forum for public debate, its extensive user-generated content renders it a valuable avenue for delving into a diverse array of social matters (17, 32). As a prominent social media venue and a rich source of text-based user discourse, Weibo also serves as a primary data source for this study and a useful source for measuring the expressed user agenda (39).

Our analysis centers on the official Weibo account of *People's Daily*, operated by the national newspaper and the CCP's central mouthpiece. Exemplifying the transformation of state-run media, the *People's Daily* joined Weibo in 2012 with the slogan “participate in, communicate and record the times”, and has acquired over 158 million followers by July 2026. As a quintessential case of cultivating cultural affinity, this party newspaper has expanded the public circulation of state-media content and created an important venue for interaction between official communication and user commentary, serving as a key source of the official agenda (15).

We used a Python-based API to retrieve relevant posts on the family planning policy, along with their subsequent comment threads, from January 2013 to June 2025. We searched for keywords including: “二胎” (second infant), “三孩” (third child) and “生育” (fertility/birth). The resulting corpus comprised 126 *People's Daily* posts and 196,218 associated user comments. For each communication event, the *People's Daily* post constituted one media document, while its associated comments were aggregated into one user document. The final analytical corpus therefore contained 252 documents organized into 126 paired communication events, totaling over 4.5 million words.

#### 3.1.1 Identification of account types

To clarify the composition of the commenters, we conducted an account-level analysis using publicly observable account metadata, including user IDs, verification status, verification descriptions and follower counts. User IDs were used to identify and deduplicate commenter accounts, resulting in 48,005 unique accounts. Of these, 3,151 displayed a verified status, accounting for 6.56% of all unique accounts, whereas 44,854 accounts, or 93.44%, did not display a verified status. Verification descriptions were used to identify observable institutional, professional, public-figure, and platform-creator affiliations, while follower counts were used as an indicator of potential audience reach. Verification status alone, however, was not treated as sufficient evidence of institutional or elite identity. On Weibo, institutions and public figures typically obtain public verification to authenticate their identities or affiliations. However, individual users may also apply for verification based on their occupations, professional qualifications, or other personal attributes. Consequently, some verified personal accounts have relatively small audiences and no observable influence.

We therefore combined verification descriptions, account names, and follower counts to classify the accounts. Verified accounts were grouped into three broad categories: institutional accounts, professional elites or public figures, and platform-certified content creators or online influencers. Institutional accounts included government agencies, news organizations, companies, educational and medical institutions, and social organizations. Professional elites and public figures included scholars, journalists, medical and legal professionals, corporate executives, and cultural or entertainment figures. Platform-certified creators included entertainment and topical bloggers, original-video creators, article authors, local bloggers, topic moderators, and certified fan-community leaders.

Importantly, we did not automatically exclude every verified account. A personal verified account was retained in the ordinary-user sample if it had fewer than 10,000 followers and displayed no observable institutional, professional-elite, public-figure, or organizational affiliation. The 10,000-follower threshold was used as a conservative proxy for potential audience reach and access to platform-based commercialization opportunities. Accordingly, the restricted sample retained non-verified individual accounts and low-reach personal verified accounts without observable institutional, elite, public-figure, or high-influence affiliations. We refer to these accounts as “user accounts” for analytical convenience.

#### 3.1.2 Analysis period

The analysis further distinguished three non-overlapping policy phases based primarily on major institutional policy milestones, while also accounting for the temporal coverage of the dataset. Period 1, the transition to the selective two-child policy, extended from January 14, 2013, to October 28, 2015 and contained 18 events. Although the selective two-child policy was formally endorsed in November 2013, the first post directly addressed the possible relaxation and continued adjustment of the family-planning system and was therefore retained as a substantively relevant pre-announcement baseline.

Period 2, the universal two-child policy period, extended from October 29, 2015, to May 30, 2021 and contained 75 events. Period 3, the three-child and pronatalist-support period, extended from May 31, 2021, to June 9, 2025 and contained 33 events. The final label reflects the fact that policy communication during this phase increasingly moved beyond the relaxation of birth limits to emphasize subsidies, childcare provision, maternal care, women's employment protection, and other fertility-support measures. This classification captures the broader transformation of China's fertility-policy orientation: from maintaining population-control restrictions, through their gradual relaxation, to the active promotion and support of childbearing.

### 3.2 Data analysis and visualization

#### 3.2.1 Text mining

Text preprocessing and topic modeling were conducted in Python. After duplicate records, URLs, punctuation, numerical strings, and other invalid elements were removed, the Chinese texts were segmented using *Jieba*. Stop words and other non-substantive tokens were then excluded, and the remaining tokens were used to construct the corpus.

Advances in natural language processing have enabled computational methods to analyze large text corpora and identify latent thematic structures. We employed Latent Dirichlet Allocation (LDA) as a computer-assisted content analysis method because of its established utility in large-scale social media text analysis (40, 41). LDA represents each document as a probability distribution over latent topics and each topic as a probability distribution over words within a hierarchical Bayesian document-topic-word framework (39, 42). Media and user texts were pooled to train a common LDA model, ensuring that the topic probabilities estimated for the two agendas were located in the same latent topic space.

Formally, for document *d*, LDA assumes a document-specific topic distribution and a topic-specific word distribution. The probability of observing word *w* in document *d* is expressed as:


$$\begin{array}{l} P\left(w|d\right)\;=\;\overset{K}{\underset{k=1}{\sum }}P\left(w|z=k\right)P\left(z=k|d\right)=\overset{K}{\underset{k=1}{\sum }}\phi_{kw}\theta_{dk}, \end{array}$$

where *K* is the number of topics, θ_*dk*_ denotes the probability of topic *k* in document *d*, and ϕ_*kw*_ denotes the probability of word *w* under topic *k*. Both distributions are assigned Dirichlet priors:


$$\begin{array}{l} \theta_{d}\sim Dirichlet\left(\alpha \right),\varphi_{k}\sim Dirichlet\left(\eta \right). \end{array}$$

In this study, the estimated document–topic distribution **θ**_*d*_ provided the topic-probability vectors used in the subsequent agenda and network analyses.

We estimated LDA models with candidate topic numbers ranging from *k* = 2 to *k* = 12. To account for variation arising from model initialization, each candidate solution was estimated using five fixed random seeds. For each value of *k*, we calculated the mean *C*_*v*_ coherence and mean log perplexity bound across the five runs. Topic coherence assessed the semantic relatedness of the highest-probability terms within each solution, while the log perplexity bound provided a complementary indicator of model fit.

To select the number of topics using a reproducible quantitative procedure, mean *C*_*v*_ coherence and mean log perplexity bound were separately rescaled to a common range from 0 to 1 and assigned equal weights. Under the measures used here, higher coherence and a higher log perplexity bound, that is, a value closer to zero, indicated better performance. The two normalized indicators were therefore combined into an equally weighted composite score. The candidate solution with the highest composite score was retained. This procedure selected the four-topic solution (see Figure 1), which provided the best overall balance between semantic coherence and model fit among the candidate solutions. The topics were interpreted by examining their highest-probability terms and the documents exhibiting the highest probability for each topic.

**Figure 1:**
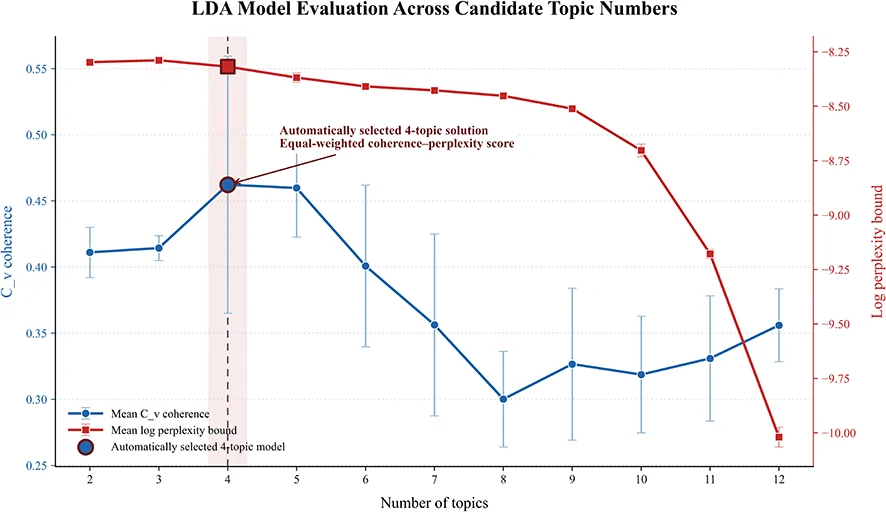
Topic coherence and log perplexity across candidate LDA models.

LDA identifies latent topics rather than theoretically predefined attributes. Based on their highest-probability terms and representative documents, we interpreted the four topics as substantive attributes of the fertility-policy agenda. Accordingly, we use the term topic when referring to LDA estimation and probability outputs, and topic-derived attribute when discussing agenda salience and network structure.

To compare the event-level topic distributions of the media and user agendas, we calculated the two-sample Kolmogorov-Smirnov statistic for each topic. We also calculated the mean probability difference as the media mean minus the user mean. A positive difference indicated greater average salience in the media agenda, whereas a negative difference indicated greater average salience in the user agenda. These comparisons were used to describe distributional divergence and average salience differences across the 126 events.

#### 3.2.2 Cross-lagged correlation analysis with the Rozelle–Campbell baseline

Agenda-setting research commonly uses cross-lagged correlations (CLC) to assess whether the salience structure of one agenda is associated with the subsequent salience structure of another. We applied the Rozelle-Campbell baseline cross-lagged correlation procedure to the topic-probability distributions of the media and user agendas (15, 43, 44). The analysis was used to identify event-lagged predictive associations and temporal precedence.

The analytical sequence was organized by communication events. The dataset comprised 126 chronologically ordered fertility-policy communication events, each consisting of a *People's Daily* post and its associated user-comment discussion. The media agendas were denoted as *X*_1_, *X*_2_, …, *X*_126_, and the corresponding user agendas as *Y*_1_, *Y*_2_, …, *Y*_126_. A one-event lag represented the transition from one communication event to the next chronologically ordered event rather than a fixed interval such as 1 day, week, or month.

This event-based specification was adopted because fertility-policy communication was irregular and concentrated around major policy announcements and related user debates. The use of fixed daily or weekly intervals would have produced numerous empty observations and potentially divided substantively connected discussions across multiple time units. Because adjacent events were not equally spaced in calendar time, the cross-lagged coefficients were interpreted as associations across successive communication events rather than as effects operating over a uniform number of days.

To describe this temporal irregularity, we calculated the elapsed time between the 125 adjacent-event pairs. The intervals ranged from 0 to 560 days, with a median of 7 days and a mean of 36.23 days. The higher mean than median indicates a positively skewed distribution resulting from a small number of long intervals. Among all transitions, 63 (50.4%) occurred within 7 days, 85 (68.0%) within 30 days, and 113 (90.4%) within 90 days. The complete distribution of inter-event intervals is presented in Figure 2.

**Figure 2:**
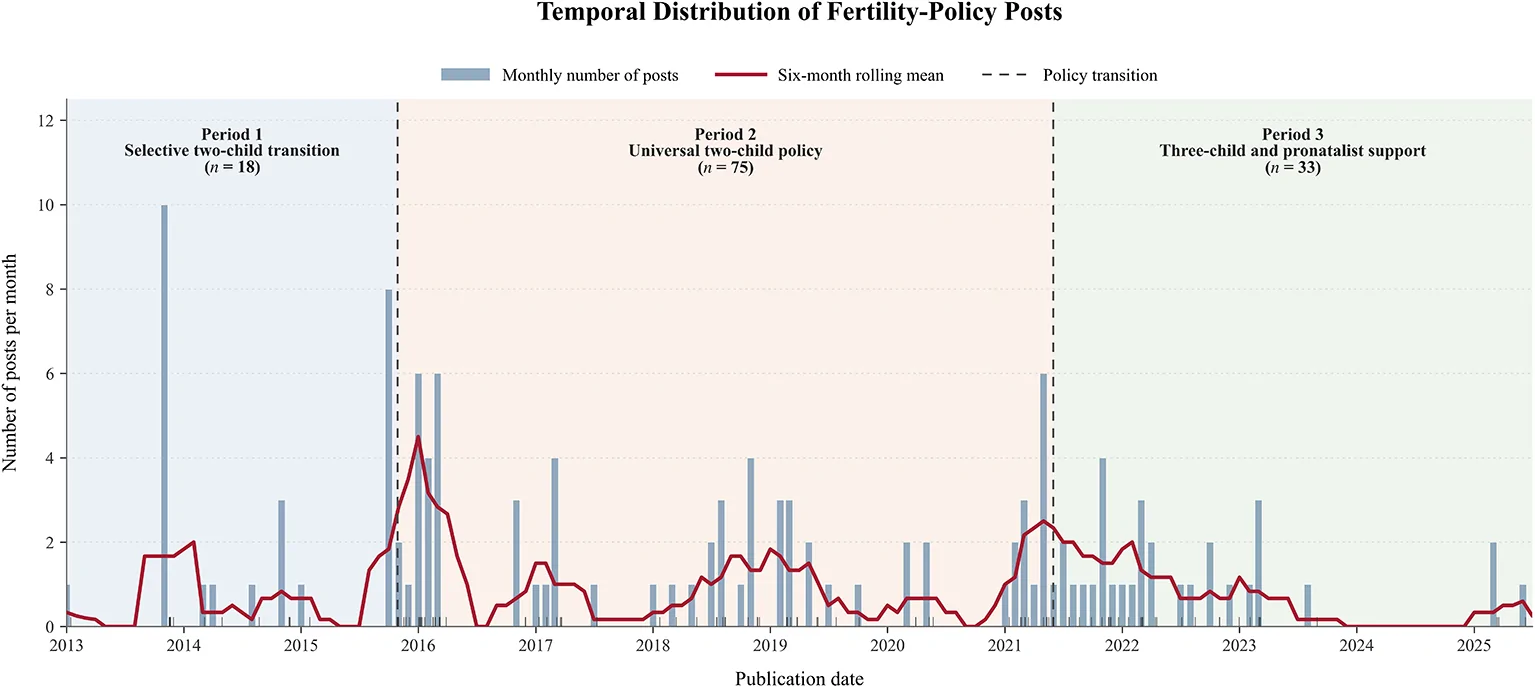
Temporal distribution of fertility-policy posts across three policy periods.

For each event *i*, *X*_*i*_ represented the media agenda as a four-dimensional topic-probability vector, and *Y*_*i*_ represented the corresponding user agenda using the same four topics. The user vector was aggregated from comments posted after the corresponding *People's Daily* post. For each pair of adjacent events, we calculated five Pearson correlations: (1) contemporaneous media-user correspondence between *X*_*i*_ and *Y*_*i*_; (2) media-agenda continuity between *X*_*i*_ and *X*_*i*+1_; (3) user-agenda continuity between *Y*_*i*_ and *Y*_*i*+1_; (4) the media-to-user event-lagged correlation between *X*_*i*_ and *Y*_*i*+1_; and (5) the user-to-media event-lagged correlation between *Y*_*i*_ and *X*_*i*+ 1_.

Each event-lagged correlation was evaluated against its corresponding Rozelle-Campbell baseline, which incorporated contemporaneous media-user correspondence and within-agenda continuity across adjacent events (45). For each adjacent-event pair, the baseline was calculated as:


$$\begin{array}{l} RC_{i}\;=\;\frac{r\left(X_{i},Y_{i}\right)+r\left(X_{i+1},Y_{i+1}\right)}{2}\times \sqrt{\frac{r{\left(X_{i},X_{i+1}\right)}^{2}+r{\left(Y_{i},Y_{i+1}\right)}^{2}}{2}} \end{array}$$

where *r*(*X*_*i*_, *Y*_*i*_) and *r*(*X*_*i*+1_, *Y*_*i*+1_) represent contemporaneous media-user correspondence at the two events, while *r*(*X*_*i*_, *X*_*i*+1_) and *r*(*Y*_*i*_, *Y*_*i*+1_) represent media- and user-agenda continuity across the adjacent events, respectively. The two directional cross-lagged correlations, *r*(*X*_*i*_, *Y*_*i*+1_) for the media-to-user direction and *r*(*Y*_*i*_, *X*_*i*+1_) for the user-to-media direction, were then compared with *RC*_*i*_.

In the primary analysis, a directional association was classified as exceeding the baseline when the difference between the corresponding cross-lagged correlation and *RC*_*i*_ was greater than 0.01. Each adjacent-event pair was subsequently classified into one of four categories: media-preceding only, user-preceding only, bidirectional, or neither. These classifications were interpreted as evidence of event-level predictive asymmetry.

#### 3.2.3 Network analysis

Network agenda-setting (NAS) analysis draws on cognitive mapping and network theory, conceptualizing an agenda as a relational structure in which the prominence of an element depends not only on its individual salience but also on its connections with other elements (46). In this study, network analysis was used at two levels. First, topic joint-intensity networks were constructed to describe how the four policy topics were organized within the media and user agendas during each policy period. Second, event-similarity networks were constructed to statistically assess the structural correspondence between the two agendas.

For the descriptive topic networks, the four topics identified through LDA constituted the network nodes. Separate networks were constructed for the media and user agendas in each of the three policy periods, producing six networks in total. Node size and color intensity represented the mean probability of each topic across the policy events included in the corresponding agenda and period. For each pair of topics, joint intensity was calculated as the mean product of their event-level probabilities:


$$\begin{array}{l} W_{ij}\;=\;\frac{1}{n}\overset{n}{\underset{e\;=\;1}{\sum }}p_{ei}p_{ej} \end{array}$$

where *p*_*ei*_ and *p*_*ej*_ denote the probabilities of Topics *i* and *j* for policy event *e*, and *n* is the number of events in the corresponding period. Larger edge weights therefore indicated that two topics tended to receive relatively high probabilities within the same policy events. Because these weights were derived from products of topic probabilities, the edges represent event-level topic joint intensity.

To statistically evaluate the structural correspondence between the media and user agendas, we constructed a second set of networks in which the 126 policy events served as nodes. For each agenda, the edge between two events was defined as the cosine similarity between their four-topic probability vectors. The resulting media and user matrices therefore represented the extent to which each pair of policy events was organized similarly in the two agendas. A higher edge value indicated that the two events had more similar topic distributions.

We then used Quadratic Assignment Procedure (QAP) correlations to compare the media and user event-similarity matrices. QAP correlation assesses correspondence between two relational matrices while accounting for the non-independence of network ties. We estimated QAP correlations for the full sample and separately for each of the three policy periods. Its basic principle is to compare the observed correlation between two matrices with correlations obtained after repeatedly randomizing the correspondence between their nodes. If the observed correlation is greater than the correlations typically produced by random permutations, the two networks are considered to exhibit statistically significant structural correspondence.

We first organized the LDA results into two topic-probability matrices, one for the media agenda and one for the user agenda. Each matrix contained the same 126 policy events as rows and the four topic probabilities as columns. For each agenda, we calculated the cosine similarity between every unique pair of events based on their four-topic probability vectors. This produced two 126 × 126 event-similarity matrices, in which each cell indicated how similarly a given pair of events was organized in the media or user agenda.

We then calculated the Pearson correlation between the corresponding event-pair similarities in the two matrices. To assess its statistical significance, we randomly permuted the event labels of one matrix 9,999 times and recalculated the matrix correlation after each permutation. A positive and statistically significant QAP correlation indicated that event pairs with similar topic distributions in the media agenda also tended to have similar topic distributions in the user agenda.

#### 3.2.4 Gender-based analysis

A gender-based analysis was conducted to examine the demographic composition and issue attention of the user agenda. Users were identified through the user ID field, and duplicate IDs were removed according to the sampling procedure described below to prevent highly active users from being counted repeatedly in the demographic analysis. Based on the resulting unique-user sample, the proportions of male and female users were calculated overall and for each policy period.

Because directly re-estimating separate LDA models for the gender-by-period subsets would have produced topic spaces that were not directly comparable with the four-topic structure identified in the main analysis, we employed a weighted dictionary-mapping procedure. The dictionary was constructed to correspond to the four LDA-derived attributes identified in the pooled corpus. Terms were assigned weights of 3 for core terms, 2 for related terms, and 1 for background terms. A longest-match-first, non-overlapping matching procedure was used to assign matched expressions uniquely. Comments were then aggregated into six gender-by-period corpora, and the weighted mapping score for each attribute was normalized by the total score across the four attributes within each corpus. The resulting normalized mapping shares were used to compare the relative issue emphasis of male and female users across the three policy periods.

## 4 Results

### 4.1 Topic-derived policy attributes and their event-level evolution

The model-selection procedure retained a four-topic solution. Based on the highest-probability terms and representative documents associated with each topic, the four topics were labeled *Policy Change and Childrearing Burdens, Women's Employment and Work-Family Responsibilities, Gender Preference and Family Norms, and Maternity Care and Childbirth Support*. These labels summarize the dominant semantic content identified by the model (see Table 1).

**Table 1:** Four latent topics identified via LDA and their top keywords.

Topic	Attribute label	Representative high-probability terms	Substantive interpretation
1	Policy Change and Childrearing Burdens	children; policy; state; second child; family planning; unable to afford; education; housing prices; pressure; fines	Policy adjustment and the economic and practical constraints on childbearing
2	Women's Employment and Work–Family Responsibilities	women; enterprises; work; maternity leave; companies; pregnancy; discrimination; employees; wages; family	Employment discrimination, maternity protection, employer costs, and gendered care responsibilities
3	Gender Preference and Family Norms	daughter; son preference; girl; son; bride price; rural areas; marriage; sex ratio; family; reproductive preference	Son preference, gender inequality, marriage practices, and family norms
4	Maternity Care and Childbirth Support	painless delivery; hospital; doctor; promotion; anesthesiologist; anesthesia; mother; woman in labor	Obstetric care, pain relief, medical access, and childbirth support

**Policy Change and Childrearing Burdens** captured discussions of changes in fertility policy and their material implications for families. Its highest-probability terms included *children, policy, state*, second *child, family planning, unable to afford, education, housing prices*, and *pressure*. The topic thus connected the relaxation of fertility restrictions with the financial and practical constraints that may discourage additionalchildbearing.

**Women's Employment and Work-Family Responsibilities** centered on the employment consequences of childbearing and the gendered distribution of paid work and caregiving responsibilities. Its prominent terms included *women, enterprises, work, maternity leave, companies, pregnancy, discrimination, employees, wages*, and *family*. This topic reflected concerns about workplace discrimination (47), employers' perceived costs of maternity protection (28), and women's simultaneous employment and caregiving responsibilities.

**Gender Preference and Family Norms** reflected gendered reproductive preferences and broader marriage- and family-related norms. Its highest-probability terms included *son preference over daughter, girl, son, bride price, rural areas, marriage, gender*, and *sex ratio*. The topic encompassed son preference, gender inequality, marriage practices, and the family norms surrounding reproductive decisions.

**Maternity Care and Childbirth Support** focused on medical and familial support during pregnancy and childbirth. Its prominent terms included *painless delivery, hospital, doctor, promotion, anesthesiologist, anesthesia, mother*, and *woman in labor*. This topic primarily captured access to labor analgesia, obstetric services, and institutional and familial support for women during childbirth.

Figure 3 presents the event-level topic probabilities of the media and user agendas across the 126 chronologically ordered communication events. The four panels correspond to the four topics identified by the LDA model, with separate lines representing the media and user agendas. The x-axis indicates the chronological event sequence, while the y-axis represents the estimated probability of each topic.

**Figure 3:**
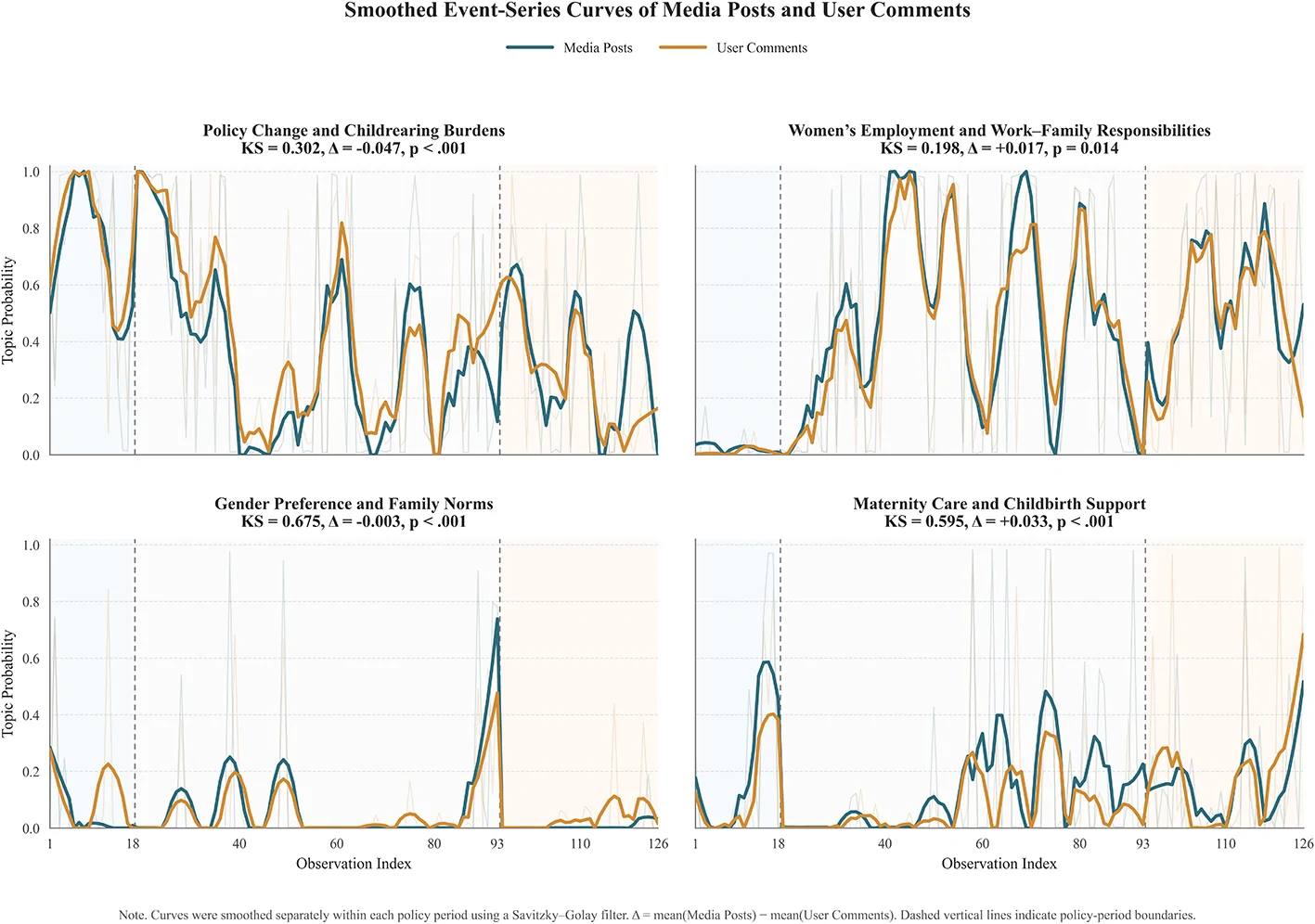
Event-level topic-probability trajectories of the media and user agendas.

Differences between the media and user distributions were summarized using the Kolmogorov-Smirnov statistic and the mean probability difference. A larger KS statistic indicates greater divergence between the two agenda distributions across the 126 events. The mean difference was calculated as the media mean minus the user mean:


$$\begin{array}{l} \Delta_{k}\;={\overset{-}{\;X}}_{k}-{\overset{-}{Y}}_{k} \end{array}$$

where ${\overset{-}{X}}_{k}$ and ${\overset{-}{Y}}_{k}$ denote the mean probabilities assigned to topic *k* in the media and user agendas, respectively. A positive value of Δ_*k*_ indicates greater average topic salience in the media agenda, whereas a negative value indicates greater average salience in the user agenda.

Figure 3 shows substantial topic-specific variation in the correspondence between the media and user agendas. *Women's Employment and Work-Family Responsibilities* exhibited the smallest distributional difference (*KS* = 0.198, *p* = 0.014), with a small positive mean difference (Δ = 0.017). This indicates that the two agendas had relatively similar overall distributions for this topic, although its average probability was slightly higher in the media agenda. *Policy Change and Childrearing Burdens* showed a larger but still moderate distributional difference (*KS* = 0.302, *p* < 0.001). Its negative mean difference (Δ = −0.047) indicates that this topic received greater average salience in the user agenda.

Larger distributional differences were observed for *Gender Preference and Family Norms* (*KS* = 0.675, *p* < 0.001) and *Maternity Care and Childbirth Support* (*KS* = 0.595, *p* < 0.001). For *Gender Preference and Family Norms*, the mean difference was close to zero (Δ = −0.003), despite the large KS statistic. This combination indicates that the media and user agendas allocated similar average attention to the topic but distributed that attention differently across communication events. In other words, similarity in average topic salience did not imply similarity in its event-level distribution. *Maternity Care and Childbirth Support* received slightly greater average attention in the media agenda (Δ = 0.033), although its event-level distribution also differed substantially from that of the user agenda.

The smoothed trajectories further reveal a broad thematic transition across the three policy periods. *Policy Change and Childrearing Burdens* was especially prominent during Period 1 but became less consistently dominant in the two later periods. In contrast, *Women's Employment and Work-Family Responsibilities* gained prominence after the transition to Period 2 and remained a major component of both agendas throughout Period 3. *Gender Preference and Family Norms* generally remained less salient and was characterized by episodic increases concentrated around particular communication events. *Maternity Care and Childbirth Support* likewise displayed event-specific fluctuations, with more visible increases during the later portions of Periods 2 and 3. Overall, the results indicate that media-user correspondence varied across topics and that similarities in mean salience could coexist with substantial differences in event-level allocation.

### 4.2 Heterogeneous event-lagged agenda correspondence across policy periods

The period-specific analysis included 123 within-period adjacent-event pairs; the two pairs spanning the boundaries between policy periods were excluded from the period-level classification. Figure 4 presents the distribution of the four event-lagged correspondence patterns at the primary RC-excess threshold of 0.01. The results reveal substantial heterogeneity across adjacent communication events and notable variation among the three policy periods.

**Figure 4:**
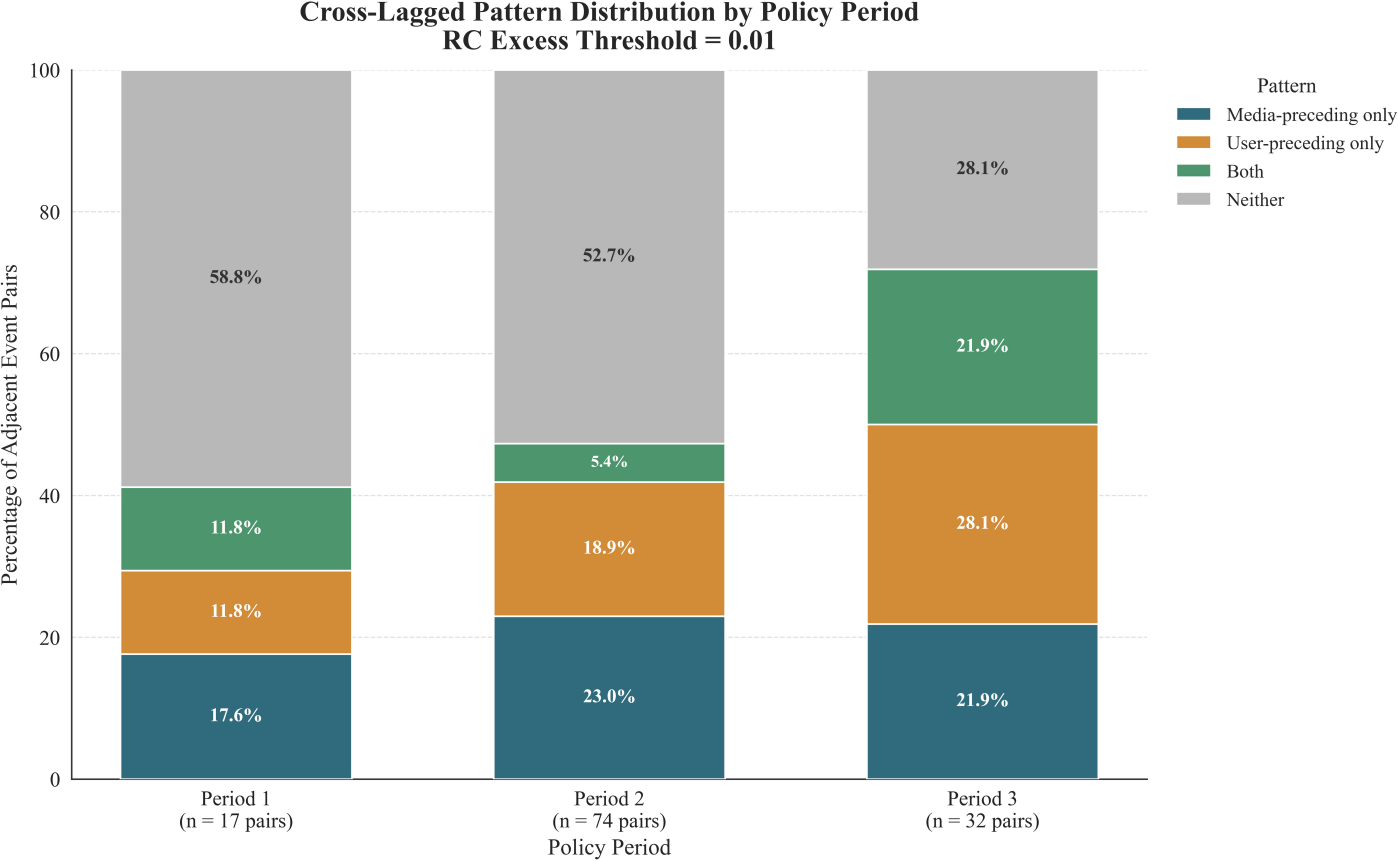
Event-lagged agenda patterns across policy periods.

In Period 1, the majority of adjacent event pairs were classified as neither direction exceeding the RC baseline (10 of 17 pairs, 58.8%). Media-preceding-only patterns occurred in three pairs (17.6%), compared with two user-preceding-only pairs (11.8%). Two additional pairs (11.8%) were classified as bidirectional. Thus, although media-preceding-only patterns were slightly more frequent than user-preceding-only patterns, most transitions provided no evidence that either directional cross-lagged correlation exceeded the RC baseline by more than 0.01.

A similar pattern was observed in Period 2, where 39 of 74 adjacent pairs (52.7%) were classified as neither. Media-preceding-only patterns occurred in 17 pairs (23.0%), whereas user-preceding-only patterns occurred in 14 pairs (18.9%). Only four pairs (5.4%) were classified as bidirectional. Period 2 therefore displayed a modest descriptive asymmetry in favor of media-preceding patterns, but this pattern was not dominant across all event transitions.

Period 3 exhibited a more differentiated distribution. The proportion classified as neither decreased to 28.1% (9 of 32 pairs), while user-preceding-only patterns increased to 28.1% (9 pairs). Media-preceding-only and bidirectional patterns each accounted for 21.9% of the pairs (7 pairs each). Relative to the first two periods, Period 3 therefore contained a substantially larger proportion of event pairs in which at least one directional coefficient exceeded the RC baseline. It was also the only period in which user-preceding-only patterns were more frequent than media-preceding-only patterns.

Taken together, the results do not indicate a uniform directional relationship across the within-period event transitions. Media-preceding-only patterns were descriptively more common than user-preceding-only patterns in Periods 1 and 2, whereas the ordering reversed in Period 3. At the same time, the persistence of neither and bidirectional classifications demonstrates considerable event-level heterogeneity.

### 4.3 Media-user network correspondence across policy periods

Figure 5 presents the topic joint-intensity networks of the media and user agendas across the three policy periods. Node size represents the mean topic probability, while edge strength represents the average joint intensity of each topic pair across the policy events within the corresponding period. The six networks were plotted using common node and edge scales to facilitate comparisons across periods and groups.

**Figure 5:**
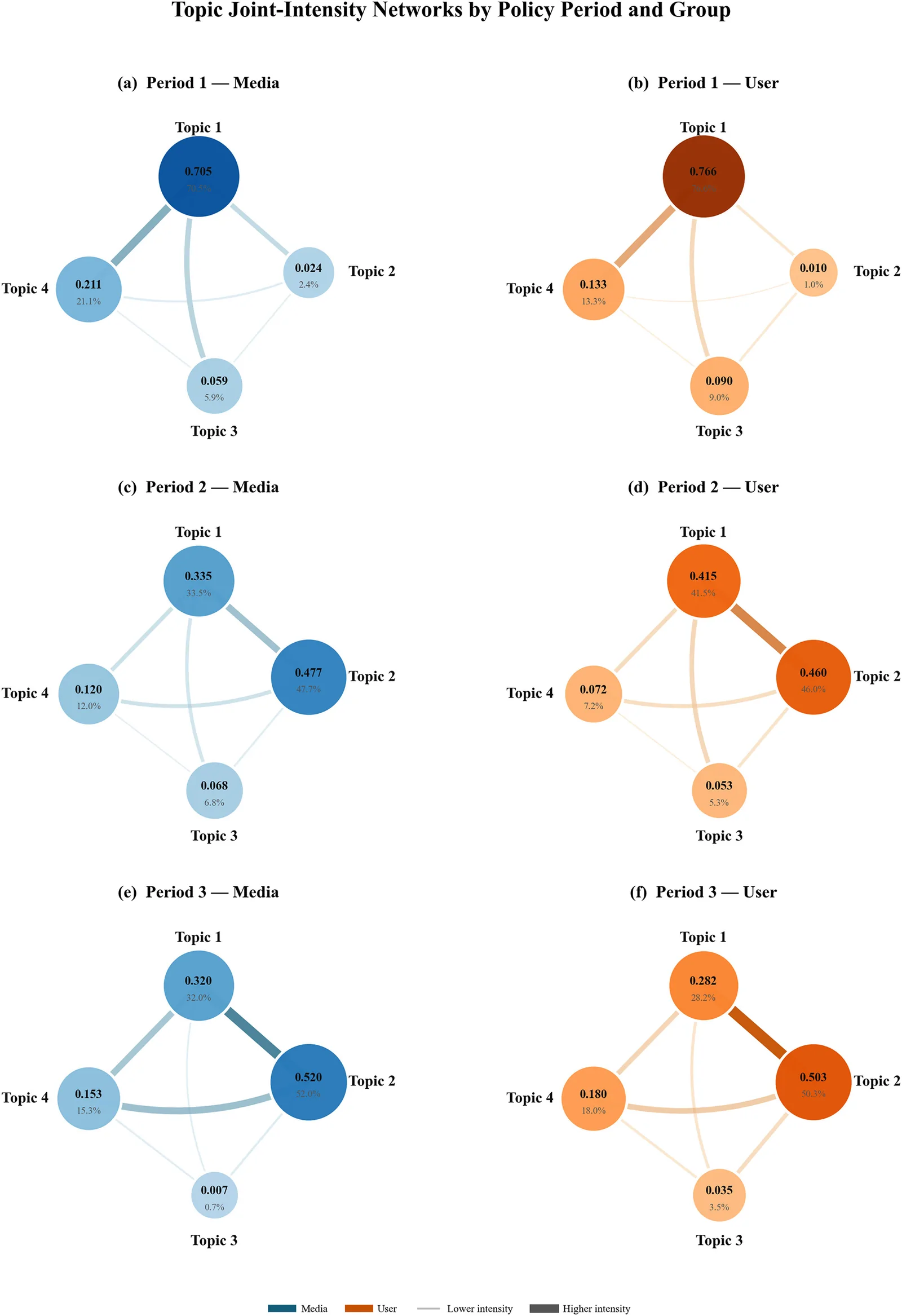
Topic joint-intensity networks of the media and user agendas across policy periods.

In Period 1, both agendas were strongly centered on *Policy Change and Childrearing Burdens*. Its mean probability was 0.705 in the media agenda and 0.766 in the user agenda. *Maternity Care and Childbirth Support* was the second most prominent topic, accounting for 0.211 of the media agenda and 0.133 of the user agenda. Correspondingly, the strongest connection in both networks was between *Policy Change and Childrearing Burdens* and *Maternity Care and Childbirth Support*, with joint intensities of 0.051 in the media network and 0.050 in the user network. Thus, the first period was characterized by a shared emphasis on policy change and its relationship with maternity and childbirth support.

In Period 2, the thematic center shifted toward *Women's Employment and Work-Family Responsibilities*, whose mean probability reached 0.477 in the media agenda and 0.460 in the user agenda. *Policy Change and Childrearing Burdens* also remained prominent, with mean probabilities of 0.335 and 0.415, respectively. The connection between these two topics became the strongest edge in both networks, with joint intensities of 0.038 for the media agenda and 0.071 for the user agenda. This indicates that policy change was increasingly discussed in conjunction with women's employment and work-family responsibilities, particularly in the user agenda.

This structure became more pronounced in Period 3. *Women's Employment and Work-Family Responsibilities* remained the dominant topic in both agendas, with mean probabilities of 0.520 in the media agenda and 0.503 in the user agenda. The strongest connection continued to be that between *Policy Change and Childrearing Burdens* and *Women's Employment and Work-Family Responsibilities*, increasing to 0.078 in the media network and 0.096 in the user network. *Maternity Care and Childbirth Support* also became more closely connected to the two dominant topics, especially in the media agenda. By contrast, *Gender Preference and Family Norms* remained peripheral in both agendas, with comparatively small node sizes and generally weak connections to the other topics.

Overall, the descriptive networks reveal a broadly similar thematic transition in the media and user agendas. Both shifted from an initial structure centered on *Policy Change and Childrearing Burdens* and *Maternity Care and Childbirth Support* toward a structure increasingly organized around the relationship between policy change and women's employment and work-family responsibilities. By Period 3, the two agendas displayed highly similar aggregate thematic centers.

Overall, the descriptive topic joint-intensity networks reveal a broadly similar thematic transition in the media and user agendas. Both shifted from an initial structure centered on *Policy Change and Childrearing Burdens* and *Maternity Care and Childbirth Support* toward one increasingly organized around the relationship between policy change and *Women's Employment and Work–Family Responsibilities*. By Period 3, the two agendas displayed comparable aggregate thematic centers.

We therefore used event-level QAP correlations to assess whether pairs of communication events exhibited corresponding topic-distribution similarities in the media and user agendas. Across the full sample of 126 events, the two event-similarity networks exhibited a strong positive correlation, *r* = 0.641, *p* < 0.001. The period-specific results nevertheless showed a non-monotonic pattern. Structural correspondence was moderately strong in Period 1, *r* = 0.551, *p* = 0.008, reached its highest level in Period 2, *r* = 0.687, *p* < 0.001, and weakened in Period 3, although it remained positive and statistically significant, *r* = 0.365, *p* < 0.001. Thus, the comparable aggregate thematic centers observed in Period 3 did not translate into stronger event-level structural correspondence.

### 4.4 Gender differences in online discourse

Figure 6 presents the gender composition of unique users overall and across the three policy periods. Among the users with valid gender information, 38.79% were male and 61.21% were female. The gender distribution also varied descriptively across periods. Female users accounted for 52.01% in Period 1, increasing to 61.34% in Period 2 and 67.39% in Period 3. Correspondingly, the proportion of male users decreased from 47.99% in Period 1 to 38.66% in Period 2 and 32.61% in Period 3. Overall, female users constituted the larger share of the unique-user sample, particularly in the later policy periods.

**Figure 6:**
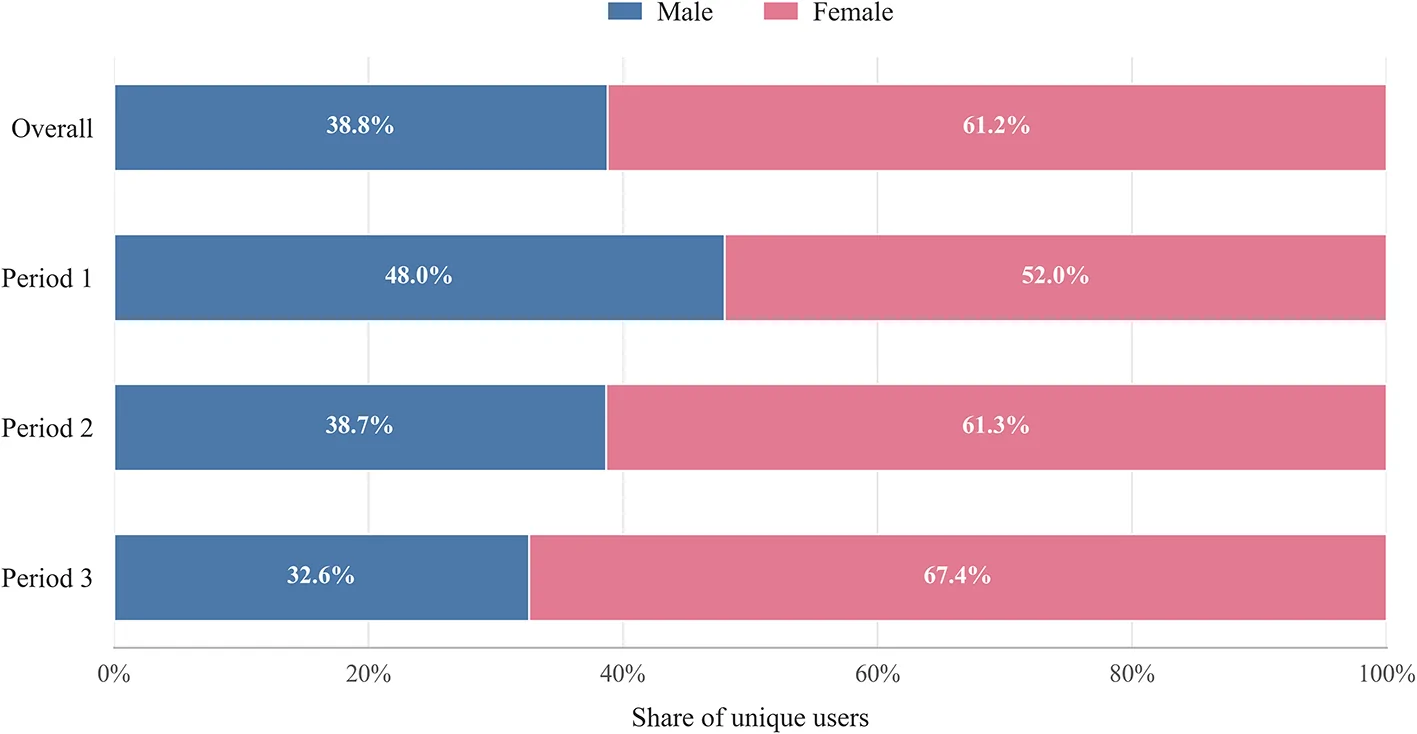
Gender composition of users across policy periods.

To examine whether this changing gender composition was accompanied by differences in issue attention, Figure 7 compares the normalized topic-mapping shares of male and female users across the three periods. Although both groups exhibited broadly similar temporal patterns, several descriptive differences emerged. Male users consistently devoted a larger share of attention to *Policy Change and Childrearing Burdens*, whereas female users showed greater attention to *Women's Employment and Work-Family Responsibilities*, particularly in the later periods. Female users also displayed higher mapping shares for *Maternity Care and Childbirth Support* across all three periods, with the widest gender gap occurring in Period 2. For *Gender Preference and Family Norms*, female users recorded higher shares in Periods 1 and 2, while male users surpassed them in Period 3.

**Figure 7:**
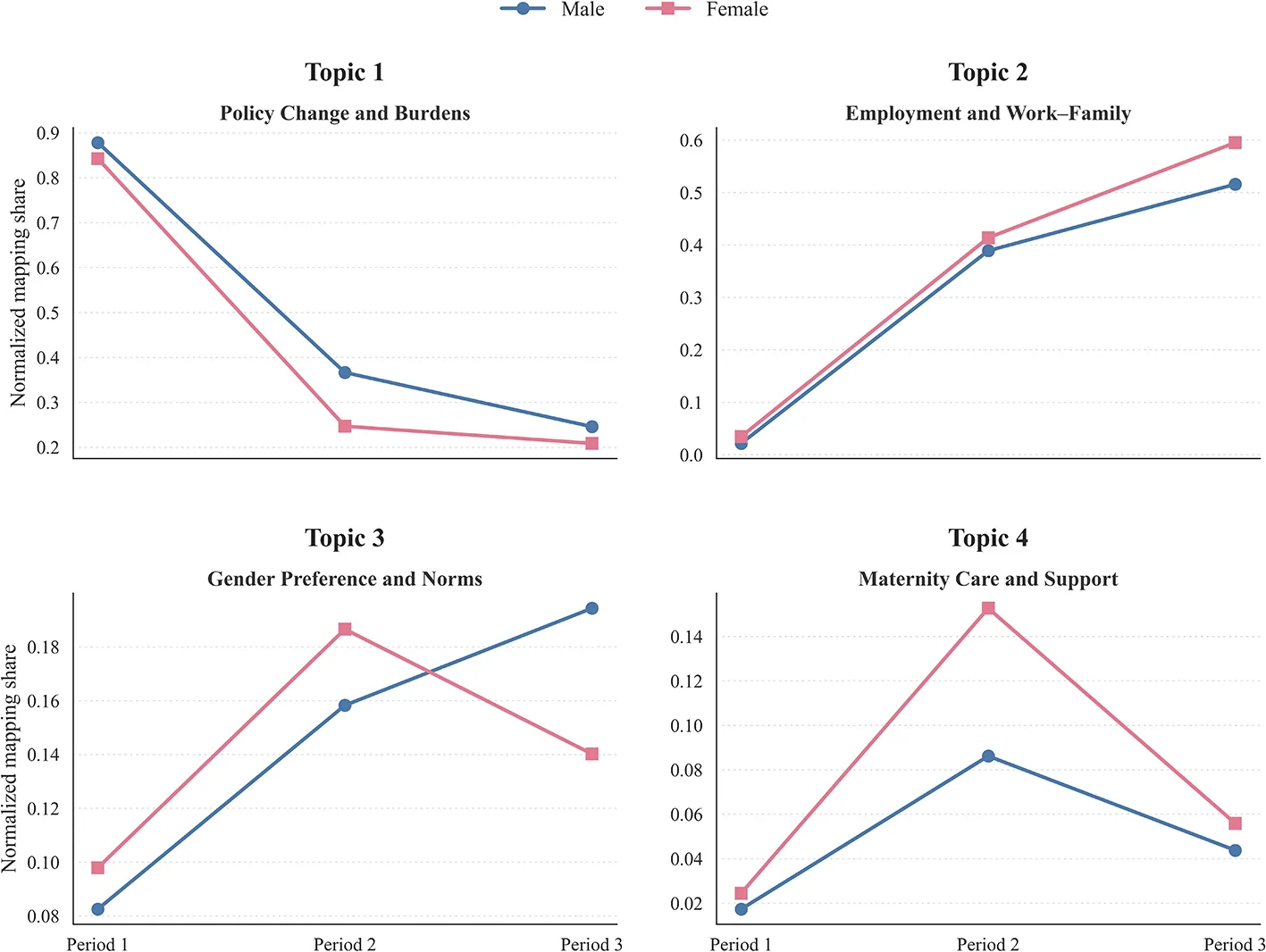
Gender differences in topic-mapping shares across policy periods.

Overall, male users' discussions were relatively more oriented toward policy changes and material burdens, whereas female users placed greater emphasis on employment, caregiving responsibilities, and maternity support. These patterns are descriptive and based on normalized weighted dictionary-mapping shares rather than LDA posterior topic probabilities, they should therefore be interpreted as differences in relative issue emphasis rather than statistically significant gender effects.

## 5 Discussion

This study advances research on online policy agenda setting by demonstrating that media-user agenda correspondence varies across analytical levels, communication events, and policy periods. First, by integrating topic salience, event-lagged correspondence, topic joint-intensity networks, and event-similarity networks, the study moves beyond analyses of isolated issues or static attribute correlations. Second, rather than examining intermedia agenda setting between news organizations (41, 48), it investigates how the state-media and user agendas developed in relation to one another on the same social media platform. Third, the 2013–2025 observation window extends beyond short-term, event-centered designs (48, 49), making it possible to examine how agenda correspondence changed as fertility policy moved from selective relaxation to broader pronatalist support.

The findings reveal substantial event-level heterogeneity: neither directional coefficient exceeded the Rozelle-Campbell baseline in most adjacent event pairs during Periods 1 and 2, although media-preceding-only patterns were descriptively more frequent than user-preceding-only patterns. In Period 3, the proportion classified as neither decreased, while user-preceding-only and bidirectional patterns became more common. These patterns indicate period-varying predictive asymmetry rather than a uniform or causal transition from media-led to user-led agenda setting.

The topic-level findings both support and complicate existing accounts of China's fertility-policy discourse. Consistent with research emphasizing the increasing individualization and social embeddedness of reproductive decision-making (32, 47), the four topics extended beyond formal policy adjustment to encompass childrearing costs, women's employment, work-family responsibilities, gender preference, family norms, maternity care, and childbirth support. The thematic emphasis of both agendas also changed over time. *Policy Change and Childrearing Burdens* was especially prominent during Period 1, whereas *Women's Employment and Work-Family Responsibilities* became increasingly central during Periods 2 and 3. This transition suggests that fertility-policy discourse developed from an initial emphasis on policy relaxation and its immediate implications toward greater attention to the institutional and gendered conditions under which additional childbearing occurs.

Media-user correspondence nevertheless differed substantially across topics. *Women's Employment and Work-Family Responsibilities* exhibited the most similar overall distributions, with only a small media advantage in mean salience. *Policy Change and Childrearing Burdens* received greater average attention in the user agenda, despite a moderate distributional difference between the two agendas. The largest divergence occurred for *Gender Preference and Family Norms*, even though its mean salience was nearly identical in the media and user agendas. This contrast is theoretically important: similar average levels of attention do not necessarily imply that the two agendas emphasize a topic during the same communication events. *Maternity Care and Childbirth Support* likewise displayed substantial event-level divergence, despite receiving only slightly greater average attention in the media agenda.

The results reveal topic-specific differences in both the level and timing of attention. The user agenda assigned greater average salience to *Policy Change and Childrearing Burdens*, whereas the media agenda devoted slightly greater average attention to *Women's Employment and Work-Family Responsibilities* and *Maternity Care and Childbirth Support*. For *Gender Preference and Family Norms*, the two agendas exhibited almost identical mean salience but markedly different event-level distributions. This finding is particularly important because it demonstrates that an apparent agreement in aggregate attention can coexist with substantial differences in when a topic becomes salient. The observed asymmetries are therefore better interpreted as differential agenda emphasis and event-level allocation than as evidence of deliberate institutional avoidance.

The period-specific event-lagged results also complicate binary accounts of agenda setting as either predominantly top-down or increasingly bottom-up. In Periods 1 and 2, media-preceding-only patterns were descriptively more frequent than user-preceding-only patterns, although neither directional coefficient exceeded the Rozelle–Campbell baseline in most adjacent event pairs. In Period 3, user-preceding-only and bidirectional patterns became more frequent, while the proportion classified as neither declined. These changes indicate period-varying temporal ordering and predictive asymmetry.

We therefore interpret the findings as evidence of significant but period-varying structural correspondence, rather than steadily increasing discursive convergence or the causal displacement of the media agenda by user discourse. This distinction avoids characterizing Chinese state-public communication as either purely propagandistic or fully participatory and instead points to a heterogeneous relationship whose direction and structural correspondence vary across events, policy periods, and levels of analysis.

The network results further demonstrate the importance of separating aggregate thematic organization from event-level structural correspondence. The descriptive topic joint-intensity networks showed that both agendas underwent a broadly similar thematic transition. By Period 3, both were centered primarily on *Women's Employment and Work–Family Responsibilities* and its relationship with *Policy Change and Childrearing Burdens*. However, the event-level QAP results followed a non-monotonic pattern: structural correspondence increased from Period 1 to Period 2 but declined substantially in Period 3, although it remained statistically significant. Comparable aggregate thematic centers therefore did not imply that the two agendas organized individual communication events in increasingly similar ways.

Several mechanisms could account for the observed structural correspondence. Media content and user comments may have responded to the same policy announcements, demographic developments, socioeconomic conditions, or other external events. Correspondence may also reflect the shared communication setting because each user-agenda document consisted of comments responding to a sampled *People's Daily* post. Reciprocal agenda adjustment is another possible explanation, but neither QAP nor the event-lagged analysis can distinguish it from these common-context mechanisms.

The findings also have direct relevance to public health policy because fertility governance extends beyond the formal relaxation of birth restrictions. Its implementation is embedded in reproductive and maternal health services, employment protection, childcare provision, household economic security, and the gendered distribution of caregiving. From this perspective, the four topics identify distinct conditions through which users may understand and evaluate fertility policy. *Policy Change and Childrearing Burdens* concerns the material feasibility of raising additional children; *Women's Employment and Work–Family Responsibilities* concerns the employment and caregiving consequences associated with childbearing; *Gender Preference and Family Norms* concerns the normative environment in which reproductive decisions are made; and *Maternity Care and Childbirth Support* concerns access to and experiences of maternal-health and obstetric services. These are therefore not only communication themes but also policy domains relevant to the implementation of fertility and reproductive-health policy.

The topic-specific results suggest correspondingly differentiated communication priorities. The greater average prominence of *Policy Change and Childrearing Burdens* in the user agenda indicates that communication focused primarily on changes in fertility rules may leave material concerns about childcare, education, housing, and family costs insufficiently addressed. The increasing centrality of *Women's Employment and Work-Family Responsibilities* in both agendas during Periods 2 and 3 highlights the importance of communicating concrete information about employment protection, maternity leave, employer obligations, childcare services, and the distribution of caregiving responsibilities. Public health communicators should therefore monitor event-specific user concerns and provide timely, service-oriented information rather than relying exclusively on generalized pronatalist messaging.

Across the three periods, the male-labeled corpora contained relatively larger mapping shares for *Policy Change and Childrearing Burdens*, whereas the female-labeled corpora contained relatively larger shares for *Women's Employment and Work–Family Responsibilities* and *Maternity Care and Childbirth Support*. The mapping pattern for *Gender Preference and Family Norms* varied across periods, with higher shares in the female-labeled corpora during the first two periods and in the male-labeled corpus during the third. These descriptive patterns are consistent with the gendered distribution of the employment, caregiving, and bodily consequences associated with fertility policy. They suggest that public health communication should not treat user concerns as homogeneous and should provide gender-sensitive information concerning workplace protection, caregiving support, reproductive health, and maternity services.

## 6 Conclusion

Taken together, these findings have practical implications for fertility-policy communication in China and comparable contexts. The event-lagged results show that the relationship between state-media and user agendas was heterogeneous rather than consistently media-led or user-led. Media-preceding-only patterns were descriptively more common in the first two policy periods, whereas user-preceding-only patterns became more frequent in the third period. However, a large proportion of adjacent event pairs, particularly in the first two periods, were classified as neither direction exceeding the Rozelle-Campbell baseline, and bidirectional patterns were also present. Policy communicators should therefore avoid assuming a stable direction of agenda leadership. Instead, they may benefit from monitoring when and around which policy events user attention diverges from or precedes official communication, particularly regarding childrearing burdens, women's employment, work-family responsibilities, gender and family norms, and childbirth support.

The gender analysis further highlights the importance of recognizing heterogeneity within the user agenda. Female users constituted an increasingly large share of participants across the three periods and placed relatively greater emphasis on *Women's Employment and Work–Family Responsibilities* and *Maternity Care and Childbirth Support*, whereas male users consistently devoted more attention to *Policy Change and Childrearing Burdens*. Attention to *Gender Preference and Family Norms* also varied by both gender and period. These descriptive patterns suggest that fertility-policy communication should account for the unevenly gendered employment, caregiving, and bodily consequences of pronatalist policies rather than treating user opinion as homogeneous.

The network results further indicate that aggregate thematic similarity should not be equated with uniform event-level convergence. Although the media and user agendas underwent a broadly similar transition from an early emphasis on policy change and maternity support toward greater attention to *Women's Employment and Work-Family Responsibilities*, their event-level structural correspondence did not increase monotonically. QAP correspondence was strongest in the second period and weakened in the third, despite the similarity of the two agendas' aggregate thematic centers during that later phase. Social media provides an observable communication environment in which state-media content and user discourse can be compared, but the degree of correspondence varies by topic, communication event, policy period, and level of analysis. The present results do not establish whether the observed correspondence reflects institutional feedback, reciprocal adjustment, or shared responses to external developments.

This study contributes to network agenda-setting scholarship by providing a thirteen-year, event-based account of how state-media and user agendas developed in relation to one another over the course of an evolving policy. Rather than treating agenda-setting power as a fixed property of either institutional media or the user, the findings reveal substantial event-level heterogeneity, period-varying predictive asymmetry, significant but non-monotonic structural correspondence, and descriptively gendered patterns of issue attention. The results should therefore not be interpreted as evidence of a causal transition from top-down media control to bottom-up user agenda setting. Instead, they indicate a dynamic relationship in which temporal precedence, structural correspondence, and gender-differentiated issue emphasis vary across successive communication events and policy stages. By integrating LDA-derived topic salience, event-driven cross-lagged analysis, topic joint-intensity networks, event-level QAP tests, and gender-based topic mapping, this study offers a transferable framework for examining longitudinal agenda relationships in other policy domains while leaving the underlying causal and institutional mechanisms for future research.

## 7 Limitations

This study has several limitations. First, the data were drawn from a single platform and one state-media account. Although Weibo is an influential venue for public discussion in China, it represents only one component of an increasingly fragmented social media environment. Moreover, *People's Daily* is a prominent central state-media organization, but its communication practices may not represent those of local governments, other state-media outlets, commercial news organizations, or policy agencies. The user agenda examined here should therefore be understood as the agenda expressed by users commenting on selected *People's Daily* posts, rather than as a comprehensive representation of Chinese public opinion. Future research could compare multiple official accounts and extend the analysis to platforms such as Douyin, WeChat, and Redbook, whose user populations, content formats, and recommendation mechanisms may produce different agenda structures.

Second, the event sample was conditioned by the study's retrieval strategy and communication setting. The analysis included *People's Daily* posts identified through a specified set of fertility-policy keywords, meaning that relevant discussions using alternative terminology may not have been captured. In addition, each user-agenda document consisted of comments responding to a corresponding *People's Daily* post. The media agenda therefore helped define both the occurrence of an event and the immediate discursive context in which the user comments were produced. This design provides a strong basis for examining media-user correspondence within shared communication episodes, but it cannot capture user discussions that emerged independently of *People's Daily* or determine whether public concerns outside these comment threads preceded official attention. Future studies could incorporate platform-wide posts, search trends, independent discussion threads, and content from multiple media sources to construct a less media-conditioned measure of the public agenda.

Third, the cross-lagged correlation analysis was based on an event clock rather than equally spaced calendar intervals. A one-event lag represents movement from one relevant communication episode to the next, while the elapsed time between adjacent observations ranged considerably. The estimated relationships should therefore be interpreted as associations across successive communication events rather than effects operating over a constant number of days. Moreover, each event-level correlation was calculated across a four-dimensional topic-probability vector. The small number of topic dimensions and the compositional nature of LDA probabilities, which sum to one within each document, may make the correlations sensitive to individual topic values and induce negative dependence among topics.

Fourth, temporal precedence and structural correspondence do not establish causal influence. Because user comments were posted after their corresponding *People's Daily* posts, contemporaneous user attention was partly conditioned by exposure to the media content. Conversely, an association between comments on one event and media content in the next event does not demonstrate that the media organization observed or responded to those comments, both agendas may have reacted to the same policy announcement, demographic development, or external event. Similarly, QAP correlations quantify correspondence between media and user event-similarity matrices but do not identify the mechanisms producing that correspondence. References to media-preceding, user-preceding, bidirectional, or reciprocal patterns therefore denote temporal ordering and predictive association, not causal effects or documented institutional responsiveness. Experimental or quasi-experimental designs, higher-frequency longitudinal data, process tracing, and direct evidence of editorial or governmental monitoring would be necessary to identify the underlying causal mechanisms.

Finally, the observed user discourse may have been shaped by platform governance, content moderation, deletion, self-censorship, and differential comment visibility. Although fertility policy has generally permitted more open discussion than highly sensitive political issues, moderation practices may still affect which comments remain observable and which comments receive prominent placement (16). The available data cannot recover deleted or unposted content, distinguish moderation from voluntary deletion, or determine how ranking algorithms affected users' exposure to particular comments. Meanwhile, as the population continues declining, particularly since China incorporated population security into the national security framework in 2024, comment moderation has intensified, on the *People's Daily*'s SNS accounts, for instance, favorable stances are promoted, with comments curated to foreground positive responses (50). Consequently, the measured user agenda represents the surviving and observable discourse within *People's Daily* comment sections rather than the full range of public attitudes. Claims about changes in moderation intensity should also be treated cautiously unless supported by direct longitudinal evidence. Future research could compare archived and live comments, examine multiple account and platform environments, and combine computational analysis with interviews or qualitative process tracing to better assess how platform governance shapes observed agenda relationships.

## Data Availability

The datasets presented in this article are not readily available because Raw Weibo data are not publicly available due to privacy and platform restrictions. De-identified aggregate data and derived analytical materials are available from the corresponding author upon reasonable request, subject to applicable ethical restrictions. Requests to access the datasets should be directed to dengwenjoy@outlook.com.
